# EmbB and EmbC regulate the sensitivity of *Mycobacterium abscessus* to echinomycin

**DOI:** 10.1002/mlf2.12139

**Published:** 2024-09-30

**Authors:** Jing He, Yamin Gao, Jingyun Wang, H. M. Adnan Hameed, Shuai Wang, Cuiting Fang, Xirong Tian, Jingran Zhang, Xingli Han, Yanan Ju, Yaoju Tan, Junying Ma, Jianhua Ju, Jinxing Hu, Jianxiong Liu, Tianyu Zhang

**Affiliations:** ^1^ Institute of Physical Science and Information Technology Anhui University Hefei China; ^2^ State Key Laboratory of Respiratory Disease Guangzhou Institutes of Biomedicine and Health, Chinese Academy of Sciences Guangzhou China; ^3^ Guangdong‐Hong Kong‐Macao Joint Laboratory of Respiratory Infectious Diseases Guangzhou Institutes of Biomedicine and Health, Chinese Academy of Sciences Guangzhou China; ^4^ China‐New Zealand Joint Laboratory on Biomedicine and Health Guangzhou Institutes of Biomedicine and Health, Chinese Academy of Sciences Guangzhou China; ^5^ University of Chinese Academy of Sciences Beijing China; ^6^ School of Pharmacy, Institute of Marine Drug Guangxi University of Traditional Chinese Medicine Nanning China; ^7^ CAS Key Laboratory of Tropical Marine Bio‐Resources and Ecology RNAM Center for Marine Microbiology, Guangdong Key Laboratory of Marine Materia Medica, South China Sea Institute of Oceanology, Chinese Academy of Sciences Guangzhou China; ^8^ School of Life Sciences University of Science and Technology of China Hefei China; ^9^ State Key Laboratory of Respiratory Disease Guangzhou Chest Hospital Guangzhou China

**Keywords:** echinomycin, EmbB, EmbC, functional compensation, *Mycobacterium abscessus*

## Abstract

Treatment of *Mycobacterium abscessus* (Mab) infections is very challenging due to its intrinsic resistance to most available drugs. Therefore, it is crucial to discover novel anti‐Mab drugs. In this study, we explored an intrinsic resistance mechanism through which Mab resists echinomycin (ECH). ECH showed activity against Mab at a minimum inhibitory concentration (MIC) of 2 µg/ml. A ΔembC strain in which the *embC* gene was knocked out showed hypersensitivity to ECH (MIC: 0.0078–0.0156 µg/ml). The MICs of ECH‐resistant strains screened with reference to ΔembC ranged from 0.25 to 1 µg/ml. Mutations in EmbB, including D306A, D306N, R350G, V555I, and G581S, increased the Mab's resistance to ECH when overexpressed in ΔembC individually (MIC: 0.25–0.5 µg/ml). These EmbB mutants, edited using the CRISPR/Cpf1 system, showed heightened resistance to ECH (MIC: 0.25–0.5 µg/ml). The permeability of these Mab strains with edited genes and overexpression was reduced, as evidenced by an ethidium bromide accumulation assay, but it remained significantly higher than that of the parent Mab. In summary, our study demonstrates that ECH exerts potent anti‐Mab activity and confirms that EmbB and EmbC are implicated in Mab's sensitivity to ECH. Mutation in EmbB may partially compensate for a loss of EmbC function.

## Impact statement

*Mycobacterium abscessus* (Mab) is characterized by rapid growth and inherent resistance to most drugs. Our research reveals that echinomycin (ECH) shows significant anti‐Mab activity, and the mechanism of resistance to ECH involves EmbB and EmbC enzymes. These enzymes modulate Mab's sensitivity to ECH by altering the permeability of the bacterial cell walls. Notably, EmbB and EmbC demonstrate a functional complementary evolution in regulating sensitivity to ECH. Collectively, our findings lay the scientific groundwork for devising effective strategies to combat Mab infections and show that ECH is a promising anti‐Mab therapeutic candidate.

## INTRODUCTION

The incidence of nontuberculous mycobacteria (NTM) infections is on the rise globally^1^. Diseases associated with NTM present significant therapeutic challenges due to the intrinsic resistance of these bacteria to many conventional antibiotics^2^. Among the most prevalent NTM, *Mycobacterium abscessus* (Mab) can cause a diverse array of infections in humans, affecting the lungs, skin, soft tissues, central nervous system, and eyes^3^, ^4^. Mab demonstrates inherent resistance to many antibiotics, including front‐line antituberculosis agents. The sensitivity of various Mab strains to different antibiotics can vary considerably, which complicates the treatment of diseases attributed to Mab infection^5^. In clinical settings, this often leads to a scarcity of effective therapeutic options and a high rate of treatment failure^6^. Consequently, there is an urgent need for a comprehensive understanding of the bacteria's resistance mechanisms and the development of potent anti‐Mab drugs for therapeutic use.

The cell wall serves as the interface between the external environment and the internal cellular components, performing many important biological functions, including maintaining structural integrity, providing protection, and facilitating transport. The cell wall is crucial for bacterial survival. Consequently, numerous enzymes involved in bacterial cell wall biosynthesis have gained attention as potential targets for drug development.

The mycobacterial *embCAB* operon encodes arabinosyltransferases, which play a role in the biosynthesis of arabinogalactan (AG) and lipoarabinomannan (LAM)^7^, ^8^. AG and LAM are key components of the mycobacterial cell wall^9^, ^10^. Proteins EmbA and EmbB are involved in synthesizing the galactan and mannan regions of AG and LAM^11^. They recognize galactose and mannose sugar molecules and transfer them onto growing polysaccharide chains^12^. EmbC is a transmembrane protein that plays a crucial role in extending arabinan chains in both AG and LAM^13^. Ethambutol (EMB), a first‐line antituberculosis drug, mediates the addition of arabinose units to the growing polysaccharide chains, thereby determining the length and structure of the arabinan^14^. Inhibiting arabinan synthesis eliminates the mycolic acid anchoring site, thereby reducing the accumulation of mycolic acids in the cell wall. Ultimately, this damages the cell wall's integrity and leads to cell death^15^, ^16^.

Mutations in the *embC*, *embB*, and *embA* genes have been associated with the resistance of *M. tuberculosis* (Mtb) to EMB^17^. The mechanism responsible for Mab's intrinsic resistance to EMB is related to polymorphisms in the *embB* gene—specifically, the substitution of Q for I at position 303 and M for L at position 304 of the arabinosyltransferase EmbB^18^. Recently, our lab reported that knocking out the *embC* gene results in the synthesis of lipomannan (LM) but not LAM, leading to increased cell membrane permeability in Mab and making it susceptible to many drugs that were originally ineffective^19^. However, the specific mechanism behind this alteration in Mab has not been clearly explained.

Studies have discovered a parallel process in *Mycobacterium smegmatis* that could be instructive. The absence of LM is not crucial for maintaining capsule integrity, but the lack of LAM can weaken the structural integrity of peptidoglycan cell walls, causing them to lose structural stability and develop “bubble‐like beads,” indicating that the cell wall is no longer firm and unable to maintain the cell's shape to resist swelling pressure^20^. Taken together, these observations suggest that EmbCAB proteins are good targets for potential antimycobacterial drugs.

A recent study demonstrated that echinomycin (ECH) shows antimycobacterial activity against Mtb H37Rv at a minimum inhibitory concentration (MIC) of 0.5 µg/ml and is even more potent against *M. bovis* (MIC: 0.1 µg/ml)^21^. However, the activity of ECH against NTM and the mechanism by which NTM resists ECH remain largely unexplored. In this study, we discovered that ECH showed potent antibacterial activity against Mab (MIC: 2 µg/ml). The resistance mechanism identified is associated with the *embCAB* operon. Our findings confirm that EmbB and EmbC are implicated in Mab's susceptibility to ECH, suggesting that the functional complementary evolution of these proteins may influence the sensitivity of Mab to ECH.

## RESULTS

### ECH shows potent anti‐Mab activity, especially when *embC* is knocked out

**Figure 1:**
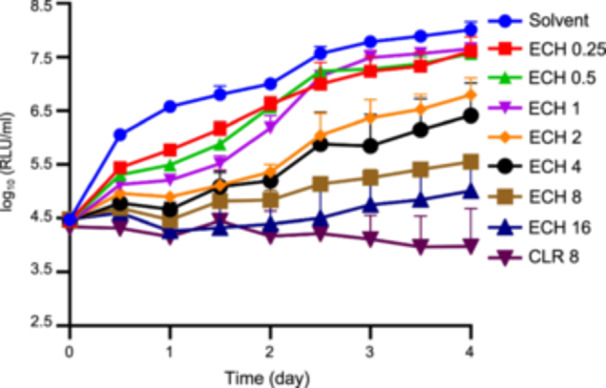
Time‐killing curves of ECH against AlMab in liquid culture. Solvent, dimethyl sulfoxide (DMSO); ECH, echinomycin; CLR, clarithromycin. The numbers after the drugs indicate the concentrations tested (μg/ml). Data are expressed as mean ± standard deviation (SD) based on three independent biological repeats. The assay was performed in triplicate in three independent experiments.

The MIC_lux_ of ECH against autoluminescent Mab (AlMab)^22^ was determined by quantifying the relative light units (RLUs). The MIC_lux_ was defined as the lowest concentration that inhibited more than 90% of the RLUs compared to a drug‐free control substance. ECH demonstrated a concentration‐dependent inhibitory effect on AlMab at an MIC of 2 μg/ml (Figure 1). To determine the sensitivity of ECH against the *embC*‐knockout Mab strain (ΔembC), the broth dilution method was used. The MIC in liquid culture (MIC_liquid_) was defined as the lowest drug concentration at which bacterial growth was not visible to the naked eye. Observations of the 96‐well plate showed that the in vitro MIC_liquid_ of ECH to ΔembC ranged from 0.0078 to 0.0156 μg/ml (Table 1).

### Mutation sites in *embB* of ΔembC mutant strains resistant to ECH

Among the spontaneously drug‐resistant mutant strains of ΔembC screened from the plates containing ECH at 0.25, 0.5, 1 or 2 µg/ml, six were randomly selected for whole‐genome sequencing (WGS), with ΔembC serving as a control. The WGS results indicated that *embB* was the most frequently mutated gene in these strains. To verify the mutations, *embB* was amplified via PCR and sequenced at Sangon Biotech (Shanghai). We identified three different mutations (G1663A, G1741A, and C1048G) in the *embB* gene of ΔembC (Table 1).

**Table 1:** MICs of ECH to ΔembC mutants and mutations in *embB* based on whole‐genome sequencing.

Strain	MIC (µg/ml)	Mutated base	Mutated amino acid
ΔembC	0.0078–0.0156	WT	WT
Mut1	0.5–1	G1663A	V555I
Mut2	1	G1741A	G581S
Mut3	0.5	C1048G	R350G
Mut4	0.25	G1741A	G581S

Mut1–4, ΔembC‐derived mutant strains after WGS; WT, wild type (no mutation).

The *embB* genes from the remaining mutants were amplified via PCR. Subsequently, the PCR products were subjected to Sanger sequencing, the results of which are presented in Table 2. Out of the 11 ECH‐resistant mutants, mutations in the *embB* gene were identified in seven strains other than those detected by WGS. Specifically, four of these seven strains showed mutations at nucleotide position 916 of the *embB* gene, leading to the substitution of D306N in the EmbB protein. In contrast, three of the seven strains had mutations at nucleotide position 917, resulting in D306 A substitution in EmbB. Notably, one strain lacked any mutations in the *embB* gene and its upstream region (approximately 200 base pairs), suggesting the potential existence of alternative ECH resistance mechanisms.

**Table 2:** MICs of ECH to ΔembC mutant strains and mutations in *embB* detected using Sanger sequencing.

Strain	MIC (µg/ml)	Mutated base	Mutated amino acid
ΔembC	0.0078–0.0156	WT	WT
Mut7^a^	0.5	G916A	D306N
Mut8	1	C1048G	R350G
Mut9^a^	0.5	G916A	D306N
Mut10^a^	1	G916A	D306N
Mut11^a^	1	G916A	D306N
Mut12	0.25	WT	WT
Mut13	1	C1048G	R350G
Mut14	0.5	C1048G	R350G
Mut15^b^	0.5	A917C	D306A
Mut16^b^	0.25	A917C	D306A
Mut17^b^	0.25	A917C	D306A

^a^ and ^b^ indicate new mutations different from those found via WGS in the strains listed in Table 1; Mut7–17, ΔembC‐derived mutant strains.

The WGS results revealed three distinct types of mutations in the *embB* gene (Table 1). Subsequent PCR identification of the remaining mutant strains unveiled two additional novel mutation types (Table 2). In total, five different mutation types (G1663A, G1741A, A917C, C1048G, G916A) were characterized in the *embB* gene of ΔembC (Table 3). We then predicted and analyzed the structure and functional domains of EmbB using InterPro and AlphaFold. Our analysis revealed that EmbB comprises three distinct domains, with the primary catalytic domain spanning amino acids 209–666, as depicted in Figure S1. Notably, all mutant sites detected (D306A, D306N, R350G, V555I, and G581S) were located in this region.

**Table 3:** Types of Mab‐*embB* mutations in ΔembC.

Type	Mutation type	
	Base mutation	Amino acid mutation
Mut‐type 1	G1663A	V555I
Mut‐type 2	G1741A	G581S
Mut‐type 3	A917C	D306A
Mut‐type 4	C1048G	R350G
Mut‐type 5	G916A	D306N

Mut‐type 1–5 indicate different mutation types.

### ΔembC mutants display variable susceptibility to different antibiotics

The broth dilution method was used to determine the MICs of various antibiotics, including moxifloxacin (MXF), levofloxacin (LEV), vancomycin (VAN), rifampin (RIF), rifabutin (RFB), ethambutol (EMB), linezolid (LZD), amikacin (AMK), cefoxitin (CEF), and clofazimine (CLF), against the ECH‐resistant mutants derived from ΔembC (Table 4). These mutants showed greater resistance to ECH than ΔembC, and the MICs of certain antibiotics, such as VAN and RIF, differed across different ΔembC mutants.

**Table 4:** MICs of different antibiotics to ΔembC mutant strains.

Antibiotics	MIC (µg/ml)						
	Mab	ΔembC	Mut‐type 1	Mut‐type 2	Mut‐type 3	Mut‐type 4	Mut‐type 5
ECH	2	0.0078	**0.5**	**1**	**0.5**	**1**	**0.5**
MXF	64	0.5	**4**	**8**	**4**	**1**	**2**
LEV	64	2	**8**	**8**	**8**	2	2
VAN	128	1	**16**	**32**	**16**	**4**	**4**
RIF	64	2	**32**	**32**	**32**	**4**	**16**
RFB	16	0.5	**2**	**4**	**4**	**1**	**1**
EMB	128	64	64	64	64	64	64
LZD	64	1	**8**	**8**	**16**	**2**	**2**
AMK	32	8	**32**	**32**	**32**	**32**	**32**
CEF	32	4	**8**	**8**	**16**	4	4

MICs were detected using the broth dilution method after incubation at 37°C for 5 days. Underlined and bold numbers represent MICs higher than the level effective against ΔembC.

### ΔembC overexpressing wild‐type and mutant *embB* genes induces resistance to different antibiotics in vitro

We observed that the strain in which the wild‐type *embB* gene was overexpressed showed no significant differences compared with ΔembC in sensitivity to a range of antibiotics, including ECH. Conversely, strains in which the mutant *embB* gene was overexpressed demonstrated increased resistance to ECH compared with ΔembC (Table 5). The differences in the MICs at which the other antibiotics countered these overexpressed mutant strains were ≤4‐fold, except for the MIC of RIF with respect to strain ΔembC::pembB^G581S^ (a ΔembC strain in which *embB* ^G581S^ is overexpressed).

**Table 5:** Sensitivity of *embB* overexpressed strains to different antibiotics.

Antibiotics	MIC (µg/ml)							
	ΔembC	ΔembC::pembB^V555I^	ΔembC::pembB^G581S^	ΔembC::pembB^D306A^	ΔembC::pembB^R350G^	ΔembC::pembB^D306N^	ΔembC::pembB^wt^	ΔembC::pVector
ECH	0.0156	0.25	0.5	0.125	0.125	0.5	0.00625	0.00625
LEV	2	2	4	2	4	8	2	2
VAN	1	2	2	2	4	4	1	1
RIF	4	4	64	8	8	8	2	2
RFB	0.5	0.25	1	2	2	1	0.5	1
EMB	64	64	128	64	64	64	64	32
LZD	1	1	2	1	2	4	1	1
AMK	8	8	8	32	8	8	4–8	16
CEF	4	8	8	16–32	8	32	8	8
MXF	0.5	0.5	1	1	1	2	0.5	0.5

ΔembC, *embC* gene knockout Mab; ΔembC::pmbB^V555I^ to ΔembC::pmbB^D306N^, *embB* ^V555I^, *embB* ^G581S^, *embB* ^D306A^, *embB* ^R350G^, *embB* ^D306N^ overexpressed in ΔembC; ΔembC::pembB^wt^, *embB* ^wt^ overexpressed in ΔembC; ΔembC::pVector represents ΔembC with pMV261A as the empty vector. MICs were detected using the broth dilution method after incubation at 37°C for 5 days.

### *embB* gene‐edited ΔembC strains are more resistant to tested drugs

The testing of the successfully *embB*‐edited strains for susceptibility to ECH and other antibiotics revealed that, in addition to a significant increase in MIC with respect to ECH, the MIC at which VAN countered the edited ΔembC was also elevated (Table 6). VAN, a glycopeptide antibiotic, inhibits cell wall synthesis.

**Table 6:** MICs of ECH and other antibiotics to *embB‐*edited strains.

Antibiotics	MIC (µg/ml)					
	ΔembC	ΔembC::embB^V555I^	ΔembC::embB^G581S^	ΔembC::embB^D306A^	ΔembC::embB^R350G^	ΔembC::embB^D306N^
ECH	0.0156	0.25	0.5	0.25	0.25	0.5
MXF	0.5	4	4	2	2	4
LEV	2	8	2	4	4	4
VAN	1	8	4	32	4	4
RIF	4	8	16	16	16	16
RFB	0.5	2	2	2	1	2
EMB	64	64	32	32	16	64
LZD	1–2	4	2	4	8	4
AMK	8	16	8	16	16	8
CEF	4	4	8	8	8	16

The MICs of different *embB*‐edited ΔembC strains were detected using the broth dilution method after incubation at 37°C for 5 days.

### ΔembC strains with mutations in the *embB* gene demonstrate resistance to ECH

**Figure 2:**
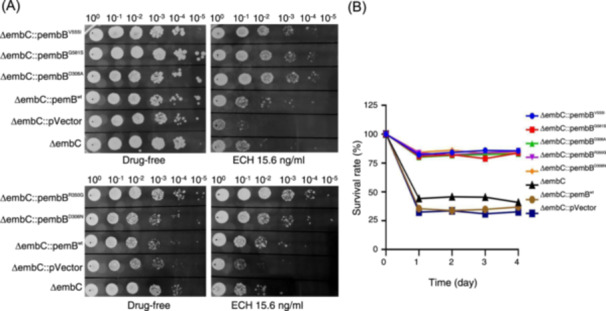
Activity of ECH to different strains in which *embB* is overexpressed. (A) Sensitivity of the overexpressed strains to ECH on plates. ΔembC, Mab with *embC* gene knockout; ΔembC::pembB^V555I^, *embB* ^V555I^ overexpressed in ΔembC; ΔembC::pembB^G581S^, *embB* ^G581S^ overexpressed in ΔembC; ΔembC::pembB^D306A^, *embB* ^D306A^ overexpressed in ΔembC; ΔembC::pembB^R350G^, *embB* ^R350G^ overexpressed in ΔembC; ΔembC::pembB^D306N^, *embB* ^D306N^ overexpressed in ΔembC; ΔembC::pembB^wt^, *embB* ^wt^ overexpressed in ΔembC. ΔembC::pVector in ΔembC with pMV261A. These strains were serially diluted, spotted on 7H10‐agar plates with or without ECH, and incubated at 37°C for 7 days. (B) Bacterial survival rates of different strains after exposure to ECH (15.6 ng/ml). All data are representative of three independent experiments.

The variable sensitivity to ECH of the ΔembC strains with overexpressed *embB* genes is presented in Figure 2. While the growth processes of different strains on the drug‐free 7H10 plate were consistent, the ΔembC strain with an overexpressed *embB* gene showed greater resistance to ECH than the ΔembC strain without *embB* overexpression.

**Figure 3:**
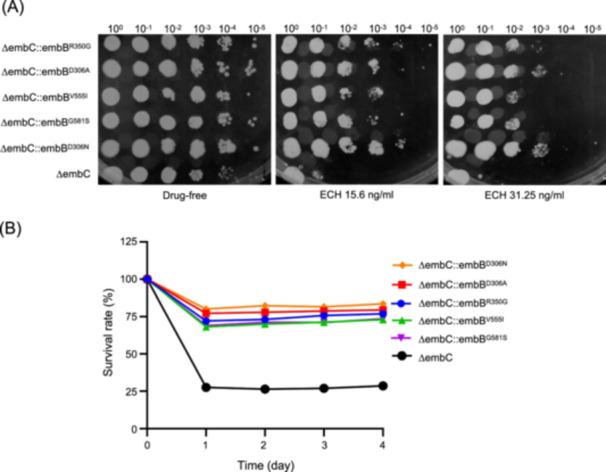
Sensitivity of different *embB* gene‐edited strains to echinomycin (ECH). (A) Sensitivity of *embB* gene‐edited strains to ECH on plates. ΔembC::embB^R350G^, *embB* ^R350G^ edited ΔembC; ΔembC::embB^D306A^, *embB* ^D306A^ edited ΔembC; ΔembC::embB^V555I^, *embB* ^V555I^ edited ΔembC; ΔembC::embB^G581S^, *embB* ^G581S^ edited ΔembC; ΔembC::embB^D306N^, *embB* ^D306N^ edited ΔembC. These strains were serially diluted, spotted on 7H10‐agar plates with or without ECH, and incubated at 37°C for 7 days. (B) Bacterial survival rates of different strains after exposure to ECH (31.25 ng/ml). All data are representative of three independent experiments.

The differences in sensitivity to ECH of ΔembC and the ΔembC strains in which *embB* was edited are presented in Figure 3. The edited ΔembC strains showed greater resistance to ECH on the plates than ΔembC, suggesting that the mutation of *embB* effectively altered the sensitivity of ΔembC to ECH.

**Figure 4:**
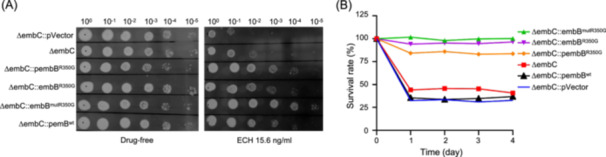
Comparison of the sensitivity of recombinant Mab strains to ECH obtained by different methods. (A) Comparison of the sensitivity of different Mab strains to ECH on plates. ΔembC::pembB^R350G^, *embB* ^R350G^ overexpressed in ΔembC; ΔembC::embB^R350G^, *embB* ^R350G^ edited ΔembC; ΔembC::embB^mutR350G^, *embB* ^R350G^ spontaneous mutant ΔembC. These strains were serially diluted, spotted on 7H10‐agar plates with or without ECH, and incubated at 37°C for 7 days. (B) Bacterial survival rates of different Mab strains after exposure to ECH (31.25 ng/ml). All data are representative of three independent experiments.

To compare the sensitivity of different strains sharing the same *embB* mutation but derived from different sources to ECH on agar plates, we selected variant *embB* ^R350G^. This selection allowed us to examine the differences in sensitivity between spontaneously resistant mutants and strains in which *embB* was edited, as well as those in which either wild‐type or mutant *embB* was overexpressed in ΔembC. As Figure 4 illustrates, the growth patterns of these various strains on 7H10 agar without drugs were uniform. However, as the concentration of ECH increased progressively, all edited ΔembC strains carrying the *embB* ^R350G^ gene showed heightened resistance to ECH. This observation underscores that an *embB* gene mutation indeed modulates the sensitivity of ΔembC to ECH. As depicted in Figure 4, the proliferation of different strains on 7H10 agar devoid of antibiotics was consistent.

### Mutated EmbB alleviates cell wall damage

**Figure 5:**
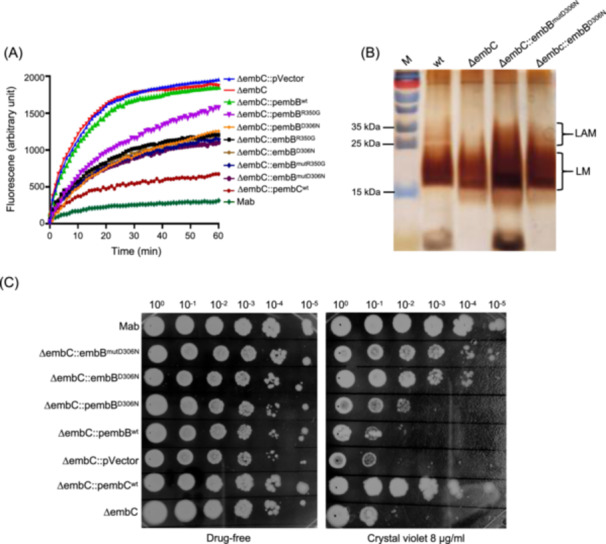
Mutated *embB* in ΔembC can alleviate cell wall damage. (A) Accumulation of ethidium bromide (EtBr) in different strains. ΔembC::pwt, *embB* ^wt^ overexpressed in ΔembC; ΔembC::pembB^R350G^, *embB* ^R350G^ overexpressed in ΔembC; ΔembC::pembB^D306N^, *embB* ^D306N^ overexpressed in ΔembC; ΔembC::embB^D306N^, *embB* ^D306N^ edited ΔembC; ΔembC::embB^mutD306N^, *embB* ^D306N^ spontaneous mutant ΔembC; ΔembC::pembC^wt^, *embC* ^wt^ overexpressed in ΔembC; Mab, wild‐type Mab. Data are expressed as mean ± SD from three independent biological repeats. The experiment was performed in triplicate (three independent experiments), and the representative results are shown. (B) Sodium dodecyl sulfate polyacrylamide gel electrophoresis analysis of LAM and LM from various strains. M: protein MW standards; wt, LAM and LM fractions from wild‐type Mab; ΔembC, LAM and LM fractions from ΔembC; ΔembC::embB^mutD306N^, LAM and LM fractions from ΔembC::embB^mutD306N^; ΔembC::embB^D306N^, LAM and LM fractions from ΔembC::embB^D306N^. (C) Sensitivity of different Mab strains to crystal violet. The experiments were performed at least three times, and only one representative image is shown in each case.

Ethidium bromide (EtBr) can permeate cells with compromised walls and subsequently bind to nuclear DNA. It emits a red–orange fluorescence when exposed to ultraviolet light. The intensity of this fluorescence is amplified in the presence of double‐stranded DNA. In an EtBr accumulation experiment, EtBr served as a substrate. By quantifying its fluorescence after cellular entry, the intracellular accumulation could be visualized, indicating the extent of the cell wall damage^23^, ^24^. Cell wall permeability studies revealed that compared to ΔembC, the accumulation of EtBr was significantly decreased in spontaneously ECH‐resistant mutant strains of ΔembC, in strains in which mutated *embB* was overexpressed, or *embB* was edited. However, the accumulated levels were still higher than those in ΔembC complemented with *embC* or in wild‐type Mab (Figure 5A). Notably, the overexpression of wild‐type *embB* in ΔembC did not result in a significant change in EtBr accumulation compared to ΔembC.

In a previous study^19^, we demonstrated that the ΔembC strain maintains LM synthesis but is deficient in LAM production, and complementary *embC* largely restores the normal phenotype. The reintroduction of *embC* largely reestablishes the wild‐type phenotype. Notably, both the ΔembC mutants and the genetically modified ΔembC strains showed partial restoration of LAM synthesis capability (Figure 5B). From these observations, we inferred that the mutant EmbB protein can partially offset the loss of EmbC function. We subsequently evaluated the sensitivity of these strains to crystal violet. As demonstrated in previous research, crystal violet, peacock green, and SDS are commonly used to assess cell walls' permeability by measuring their toxicity to mycobacteria^25^. The outcomes were consistent with those of our EtBr accumulation experiment (Figure 5C). These findings indicate that mutated EmbB has the capacity to mitigate the partial loss of EmbC function, thereby diminishing the permeability of the cell wall in ΔembC. In essence, mutated EmbB can partially restore cell wall integrity compromised by the absence of EmbC. Nevertheless, it is incapable of fully assuming the function of EmbC.

### EmbB and EmbC share conserved amino acids in Mab

In our experiment, the absence of the *embC* gene generated an unforeseen compensatory mutation in the *embB* gene, which partially compensated for the lost function of *embC*. To delve deeper into this phenomenon, the amino acid sequences of EmbB and EmbC were compared (Figure S2). The identity between EmbB and EmbC in Mab was approximately 42.23%. It is noteworthy that EmbB and EmbC shared identical amino acids at position 350 (in EmbB), where R was replaced with G, as corroborated by the WGS results. Moreover, the amino acid at position 555 in EmbB underwent a mutation into I, mirroring the corresponding residue in EmbC. No other mutations were observed to show a significant correlation with the amino acid sequence of EmbC.

### Mab EmbB mutations at conserved sites

A sequence comparison with the spontaneously drug‐resistant ΔembC mutant gene revealed that the homologous proteins in Mtb, *M. bovis*, *M. marinum*, *M. avium*, *M. ulcerans*, and *M. canettii* are all classified as arabinosyltransferases B (EmbB). The sequence alignment indicated that Mab EmbB was approximately 67% identical to EmbB across these species (Table S1). Figure S3 identifies the sites of spontaneous resistance mutations, highlighting how these sites correspond to conserved sites within mycobacterial EmbB. This suggests that such mutations could potentially alter the function of EmbB.

## DISCUSSION

Mab, the most common NTM pathogenic bacteria, is naturally resistant to various antibiotics^4^, ^26^. Only a few antibiotics, such as CLR, azithromycin, and AMK, are effective in treating certain diseases caused by Mab^27^. Consequently, the pursuit of novel drug discovery and development is imperative. Although Mab's pathogenicity has been widely studied, further investigation is necessary to elucidate the precise mechanism(s) responsible for its resistance. Understanding the functionality of Mab proteins is crucial to forestall potential threats.

Most bacteria possess a protective outer layer known as the cell wall. The low permeability of this cell wall in Mab is crucial to its intrinsic resistance^28^. The *embC* gene encodes arabinosyltransferase C, an enzyme essential for cell wall synthesis. If the function of *embC* is compromised, the synthesis of the cell wall is disrupted, enhancing its permeability. This increased permeability renders Mab more susceptible to antibiotics^19^, ^29^.

In this study, we discovered that the MIC at which ECH counters Mab is 2 µg/ml. However, this value significantly decreased to a range of 0.0078–0.0156 µg/ml when ECH was tested against the ΔembC strain. Through WGS analysis of spontaneously resistant ΔembC mutant strains, we identified mutations in the EmbB protein at positions D306A, D306N, R350G, V555I, and G581S. *embB*, an essential gene that encodes arabinosyltransferase B, plays a critical role in cell wall synthesis. Notably, overexpression of the *embB* gene in ΔembC led to increased resistance to ECH (MIC: 0.25–0.5 µg/ml, representing a 32‐ to 128‐fold increase in resistance).

Simultaneously, the *embB* gene in ΔembC was edited using a CRISPR/Cpf1‐assisted homologous recombination system that we recently developed^30^, ^31^. The edited strains also demonstrated increased resistance to ECH (MIC: 0.25–0.5 µg/ml). Furthermore, a protein sequence comparison revealed a 42.23% identity between the amino acid sequences of EmbB and EmbC. These results suggest that both *embB* and *embC* are associated with ECH resistance in Mab. Considering the involvement of both EmbB and EmbC in cell wall synthesis^29^, we hypothesized that EmbB and EmbC may alter the sensitivity of Mab to ECH by modifying its cell wall permeability. This hypothesis was confirmed by monitoring EtBr accumulation in ΔembC strains in which *embB* was overexpressed and in which it was edited. It was further supported by examining the cell wall components that demonstrated a partial restoration of LAM synthesis in the ECH‐resistant ΔembC strains. Drug‐resistant ΔembC isolates with *embB* mutations were also obtained when screened with different antibiotics, such as LZD (unpublished data). These findings suggest that when ΔembC is exposed to antibiotic stress, EmbB may mutate to compensate for the loss of partial function of EmbC, ensuring its survival.

In summary, this investigation reveals that a natural compound, ECH, shows potent inhibitory activity against Mab. Nevertheless, a comprehensive elucidation of the mechanism through which ECH exerts its activity and identifies its potential molecular targets within mycobacteria remains to be reported. We identified two genes in Mab that confer resistance to ECH, namely, *embB* and *embC*, which encode arabinosyltransferases. Notably, when EmbC is absent in Mab and unable to fulfill its physiological role, EmbB undergoes mutation under selective pressure to compensate for the loss of EmbC's function, thereby facilitating adaptation to environmental perturbations. Our findings suggest that Mab may modulate its sensitivity to ECH through functional compensatory mechanisms.

## MATERIALS AND METHODS

### Drug formulation

ECH was provided by the South China Sea Institute of Oceanology, Chinese Academy of Sciences, and dissolved in dimethyl sulfoxide (DMSO). MXF, LEV, VAN, RIF, RFB, LZD, CLR, CEF, CLF, apramycin (APR), and anhydrotetracycline (ATC) were bought from MeilunBio and dissolved in DMSO, whereas AMK (Meilunbio) and kanamycin (KAN) (Meilunbio) were dissolved in distilled water. Zeocin‐Bleomycin (ZEO) (InvivoGen).

### Strains and growth conditions

Mab, AlMab, and ΔembC were cultured in Middlebrook 7H9 broth medium containing 10% Oleic Albumin Dextrose Catalase (OADC) and 0.05% Tween 80 at 37°C for 3‐5 days until the OD_600_ reached a value between 0.6 and 0.8 or on 7H10/7H11 solid medium containing 10% OADC 37°C for 7 days. *Escherichia coli* trans‐T1 (TransGen Biotech) was cultured at 37°C in LB broth/solid medium with appropriate antibiotics.

The antibiotic concentrations (µg/ml) used for Mab and ΔembC were as follows: KAN 100, APR 230, and ZEO 30; for *E. coli*, the antibiotic concentrations (µg/ml) used were as follows: KAN 50, APR 50, and ZEO 30.

### Testing the sensitivity of ECH against Mab and ΔembC in vitro

#### In vitro activity of ECH against AlMab

AlMab was inoculated into 5 ml of 7H9 broth medium containing 10% OADC and 0.05% Tween 80. The culture was incubated at 37°C and 200 rpm until the OD_600_ reached 0.6–0.8. RLUs were measured using a luminescence detector (Promega GloMax2020™) at 12 h intervals. If the RLUs of 200 μl bacteria exceeded 2 × 10^7^, the bacterial culture was diluted with 7H9 medium without Tween 80 to achieve approximately 3000–5000 RLUs/200 µl in the diluted sample as the final tested culture.

ECH was dissolved in DMSO to achieve concentrations of 1.6, 0.8, 0.4, 0.2, 0.1, and 0.05 mg/ml, while CLR was prepared at a concentration of 0.8 mg/ml. To obtain the desired test concentrations (16, 8, 4, 2, 1, and 0.5 µg/ml), 2 µl of each ECH concentration was combined with 198 µl of diluted AlMab in separate 1.5 ml EP tubes. Each concentration was tested in triplicate. Additionally, both positive (AlMab with 8 µg/ml CLR) and negative (bacterial culture with DMSO) control groups were included in the experiment.

#### In vitro activity of ECH against ΔembC

The anti‐ΔembC activity of ECH was assessed using the broth dilution method. ΔembC was inoculated into 5 ml of 7H9 broth and incubated at 37°C with agitation at 200 rpm until the OD_600_ reached 0.6–0.8. Subsequently, the culture was adjusted to an OD_600_ of 0.125, and ECH was diluted to a concentration of 2 μg/ml using 7H9 medium without Tween 80. The final test concentrations of ECH ranged from 1 μg/ml to 0 in two‐fold serial dilutions. The diluted ΔembC cultures were then added to a 96‐well plate containing the serial drug dilutions and incubated at 37°C for 7 days.

### Screening of spontaneous ECH‐resistant mutants using ΔembC

The ΔembC was cultured in 100 ml of 7H9 liquid medium (containing 10% OADC, 0.05% Tween 80, and 5% ethyl bromide as mutagen) in a 250 ml conical flask and incubated at 37°C and 220 rpm to obtain the OD_600_ of 0.6–0.8. Subsequently, 500 µl of 10‐fold concentrated bacterial solution was plated on 7H10 agar plates containing different concentrations (0.25, 0.5, 1, 2 μg/ml) of ECH and incubated at 37°C. Three plates were set for each concentration. Each individual colony was picked and inoculated into 2 ml of 7H9 in a 50 ml tube and incubated at 37°C and 200 rpm. The liquid culture was diluted and spread to obtain pure single colonies. Then, the sensitivity of these isolated and pure colonies to ECH was detected.

### Identification of the mutant genes and mutation sites

ΔembC spontaneous resistant mutants were randomly selected and sent to Genewiz Biology Company (Suzhou) after DNA extraction for WGS. The sequencing reads of ΔembC were compared with that of the parent strain to seek meaningful mutation sites. Primers were designed to amplify and validate the corresponding mutant genes in these strains.

### Drug susceptibility testing of ΔembC mutants

The broth dilution method was used to detect the susceptibilities of ΔembC mutants to various antibiotics. Stock solutions of ECH, RIF, RFB, MXF, LEV, LZD, CEF, VAN, and AMK were prepared by diluting in 7H9 medium without Tween 80, ensuring that the final concentration of DMSO did not exceed 2%. The Mab strains were cultivated in 7H9 medium until the optical density at OD_600_ reached 0.6–1, subsequently adjusted to an OD_600_ of 0.125 using 7H9 medium without Tween 80. Subsequently, 100 μl of the diluted culture and an equal volume of the drug solution were combined in each well. The MICs were determined after a continuous incubation at 37°C for 7 days.

### Overexpression of *embB* gene in ΔembC

Six variants (*embB* ^wt^, *embB* ^V555I^, *embB* ^R350G^, *embB* ^G581S^, *embB* ^D306A^, *embB* ^D306N^) of the *embB* gene were amplified from ΔembC and spontaneous‐resistant ΔembC mutants using PCR with primers CZ‐Mab_embB‐F/CZ‐Mab_embB‐R (5′‐GGCCAAGACAATTGCGGATCCATGACAGAGAATTCCGTGACAGATAC‐3′/5′‐ACATCGATAAGCTTCGAATTCTTACGGCTTGATCCGGATCTG‐3′). These were subsequently inserted into the pMV26A plasmid under the *hsp60* promoter's control. The resulting six plasmids were then transformed into the ΔembC strain. The MICs of ECH to the recombinant strains in liquid media were determined as described above.

### *embB* gene editing in ΔembC

The editing of *embB* in ΔembC was accomplished using the CRISPR‐Cpf1 mediated homologous recombination system, as detailed previously^30^. Initially, the pJV53‐Cpf1 plasmid was introduced into ΔembC to create ΔembC::pJV53‐Cpf1, which was subsequently prepared as competent cells. Primers listed in Table S2 were used to synthesize two single‐stranded DNA fragments. These fragments were annealed and ligated with linearized pCR‐Zeo to yield pCR‐embB^mut^. A 59 bp single‐stranded DNA containing the desired mutation(s) was synthesized as a template for introducing point mutations. The five pCR‐embB^mut^ plasmids, along with their corresponding single‐stranded template DNA, were co‐transformed into the competent cells of ΔembC::pJV53‐Cpf1 via electroporation. The transformants were then plated on 7H11 medium supplemented with ATC (200 ng/ml), KAN (100 µg/ml), and ZEO (30 µg/ml) and incubated for 7 days. Upon confirmation of successful *embB* gene editing, the MICs of ECH to the edited strains were determined as described above.

### Sensitivity of different strains to ECH

The overexpressed and edited strains as well as ΔembC were cultured to an OD_600_ of 0.6. Subsequently, 2 µl of the serial tenfold diluted culture (10^0^, 10^−1^, 10^−2^, 10^−3^, 10^−4^, and 10^−5^) was dropped onto each square drawn on 7H10 plates containing varying concentrations of ECH. The plates were then incubated at 37°C for 7 days. Concurrently, bacterial cultures were diluted to an OD_600_ of 0.05 and exposed to varying concentrations of ECH in triplicate; the colony‐forming units (CFUs) were enumerated on 7H10 agar plates at predetermined time points.

To determine the sensitivity of different strains of the same mutant type to ECH, we selected a mutant type, *embB* ^R350G^. We cultured ΔembC, ΔembC::embB^mutR350G^, ΔembC::embB^R350G^, ΔembC::pVector, ΔembC::pembB^mutR350G^, and ΔembC::pembB^wt^ in 7H9 broth at 37°C and 200 rpm until the OD_600_ reached 0.6–0.8. We then dropped 2 µl of serial 10‐fold diluted cultures (10^0^, 10^−1^, 10^−2^, 10^−3^, 10^−4^, and 10^−5^) onto each square of 7H10 plates containing varying concentrations of ECH. The plates were incubated at 37°C for 7 days, and CFUs were counted on the 7H10 plates at the specified time points.

### Cell wall permeability detection

To assess the impact on cell wall permeability in ΔembC, two mutant strains, *embB* ^D306N^ and *embB* ^R350G^, were subjected to EtBr accumulation assays. The following cultures were grown until they reached an OD_600_ of 0.8: Mab, ΔembC, ΔembC::embB^R350G^, ΔembC::embB^D306N^, ΔembC::embB^R350G^, ΔembC::embB^D306N^, ΔembC::pVector, ΔembC::pembB^R350G^, ΔembC::pembB^D306N^, ΔembC::pembB^wt^, and ΔembC::pembC^wt^. After collecting the bacteria by centrifugation and washing them with PBS‐Tw (PBS supplemented with 0.05% Tween 80), the OD_600_ was standardized to 0.4. A solution of 1 mg/ml EtBr in DMSO was diluted to a concentration of 4 μg/ml in PBS‐Tw with 0.8% glucose. Subsequently, 100 μl of this diluted culture was transferred into each well of a white 96‐well plate, with three repetitions for each strain. A mixture consisting of 100 μl of a diluted culture, which contained 4 μg/ml of EtBr and 0.8% glucose, was introduced into each well. Two control groups were established: a sterile control group (combining 100 μl PBS‐Tw with 100 μl EtBr) and a non‐EtBr control group (combining 100 μl PBS‐Tw with 100 μl of diluted bacteria), with each group having three replicates. The real‐time fluorescence of the 96‐well plate was measured using a multifunctional microplate reader (PerkinElmer), with the excitation and emission wavelengths set to 530 and 590 nm, respectively. The fluorescence values from the experimental group were corrected by subtracting the baseline EtBr fluorescence values obtained from the corresponding sterile control group. This correction ensured that the fluorescence measurements from the non‐EtBr control group remained minimal and did not show an increase over time^23^, ^32^.

### Susceptibility of various Mab strains to crystal violet

We tested the toxicity of crystal violet (8 µg/ml) to various strains: Mab, ΔembC, ΔembC::embB^mutD306N^, ΔembC::embB^D306N^, ΔembC::pVector, ΔembC::pembB^D306N^, ΔembC::pembB^wt^, ΔembC::pembC^wt^. These strains were cultured until an OD_600_ of 0.8 was reached. Subsequently, 2 µl of the serial tenfold dilutions (10^0^, 10^−1^, 10^−2^, 10^−3^, 10^−4^, and 10^−5^) of the cultures was dropped onto each square drawn on 7H10 plates containing varying concentrations of crystal violet and incubated at 37°C for 7 days.

### Extraction and analysis of LAM and LM

Mab, ΔembC, ΔembC::embB^mutD306N^, and ΔembC::embB^D306N^ were cultured until the OD_600_ reached 0.8‐1. Bacteria were harvested by centrifugation and then incubated with 200 µl of a CHCl_3_/CH_3_OH mixture (1:1:0.3, v/v/v) at 55°C for 30 min. The residual material was further extracted using 200 µl of PBS‐saturated phenol and 200 µl of ddH_2_O at 80°C for 2 h after being centrifuged at 18,000*g* for 5 min. The sample was then cooled and thoroughly mixed with 100 µl of CHCl_3_. After another round of centrifugation at 18,000*g* for 15 min, the upper aqueous layer (containing LAM and LM) was transferred to a new tube without a cap. This aqueous layer (containing LAM and LM) was then micro‐dialyzed against flowing distilled water at room temperature overnight using a 3.5‐kDa MWCO membrane. Finally, the profile of LAM and LM was monitored using periodic acid/Schiff (PAS) staining after running a 15% SDS‐polyacrylamide gel^33^, ^34^.

### Bioinformatics analysis of EmbB and EmbC

Amino acid sequences of Mab‐EmbB and Mab‐EmbC were retrieved from the National Center for Biotechnology Information (NCBI) database (https://www.ncbi.nlm.nih.gov/) and subsequently compared. Further, a search for homologous proteins to MAB_0185 (MAB‐EmbB) was conducted across various mycobacteria species, including Mab, Mtb, *M. bovis*, *M. avium*, *M. marinum*, *M. canettii*, and *M. ulcerans*, Sequence alignment analysis was carried out to determine if the mutations in Mab‐EmbB were situated within conserved regions (domains).

## AUTHOR CONTRIBUTIONS

**Jing He**: Investigation (lead); methodology (equal); software (equal); validation (equal); visualization (lead); writing—original draft (lead). **Yamin Gao**: Data curation (equal); formal analysis (equal); methodology (equal); writing—original draft (supporting); writing—review and editing (equal); funding acquisition (equal). **Jingyun Wang**: Investigation (supporting); data curation (equal). **H. M. Adnan Hameed**: Data curation (equal); writing—original draft (equal); writing—review and editing (lead); funding acquisition (equal); project administration (equal); supervision (equal). **Shuai Wang**: Conceptualization (supporting); formal analysis (equal); funding acquisition (supporting). **Cuiting Fang**: Methodology (supporting); data curation (equal). **Xirong Tian**: Investigation (supporting); validation (supporting). **Jingran Zhang**: Investigation (supporting); methodology (supporting) **Xingli Han**: Investigation (supporting); software (supporting). **Yanan Ju**: Investigation (supporting); software (supporting). **Yaoju Tan**: Supervision (supporting); funding acquisition (equal). **Junying Ma**: Resources (equal); funding acquisition (equal); project administration(equal). **Jianhua Ju**: Resources (equal); data curation (equal); funding acquisition (supporting). **Jinxing Hu**: Supervision (supporting); validation (equal). **Jianxiong Liu**: Conceptualization (equal); funding acquisition (equal); project administration (equal). **Tianyu Zhang**: Conceptualization (lead); funding acquisition (lead); project administration (lead); resources (supporting); validation (equal); writing—review and editing (equal).

## ETHICS STATEMENT

This study did not involve any experiments on animals or humans.

## CONFLICT OF INTERESTS

The authors declare no conflict of interests.

## Supporting information

Supporting information.

## Data Availability

All data generated or analyzed during this study are included in this article.
