# Supplementary material for: EmbB and EmbC regulate the sensitivity of Mycobacterium abscessus to echinomycin
Source: mLife. 2024 Sep 30;3(3):459–70. doi: 10.1002/mlf2.12139 (PMC11442130; doi:10.1002/mlf2.12139)
Supplement: Supplementary file 1 — Supporting information. [file MLF2-3-459-s001.docx]

Supplementary Information

EmbB and EmbC Regulate the Sensitivity of *Mycobacterium abscessus* to Echinomycin

Jing He^1,2,3,4#^, Yamin Gao^2,3,4,5#^, Jingyun Wang^6,7^, H.M. Adnan Hameed^2,3,4,5*^, Shuai Wang^2,3,4,5^, Cuiting Fang^2,3,4,5^, Xirong Tian^2,3,4,5^, Jingran Zhang^2,3,4,8^, Xingli Han^2,3,4,5^, Yanan Ju^2,3,4,8^, Yaoju Tan^9^, Junying Ma^7^, Jianhua Ju^7^, Jinxing Hu^9*^, Jianxiong Liu^9*^, Tianyu Zhang^1,2,3,4,5,8,9*^

^1^ Institute of Physical Science and Information Technology, Anhui University, Hefei, 230601, China

^2^ State Key Laboratory of Respiratory Disease, Guangzhou Institutes of Biomedicine and Health, Chinese Academy of Sciences, Guangzhou, 510530, China

^3^ Guangdong-Hong Kong-Macao Joint Laboratory of Respiratory Infectious Diseases, Guangzhou Institutes of Biomedicine and Health, Chinese Academy of Sciences, Guangzhou, 510530, China

^4^ China-New Zealand Joint Laboratory on Biomedicine and Health, Guangzhou Institutes of Biomedicine and Health, Chinese Academy of Sciences, Guangzhou 510530, China

^5^ University of Chinese Academy of Sciences, Beijing, 100049, China

^6^ School of Pharmacy, Institute of Marine Drug, Guangxi University of Traditional Chinese Medicine, Nanning 530200, China

^7^ CAS Key Laboratory of Tropical Marine Bio-Resources and Ecology, RNAM Center for Marine Microbiology, Guangdong Key Laboratory of Marine Materia Medica, South China Sea Institute of Oceanology, Chinese Academy of Sciences, Guangzhou 510301, China

^8^ School of Life Sciences, University of Science and Technology of China, Hefei, 230027, China

^9^ State Key Laboratory of Respiratory Disease, Guangzhou Chest Hospital, Guangzhou 510095, China

^#^These authors contributed equally to this work.

^*^Correspondence: H.M. Adnan Hameed, adnan@gibh.ac.cn; Tianyu Zhang, [zhang_tianyu@gibh.ac.cn](mailto:zhang_tianyu@gibh.ac.cn); Jianxiong Liu, ljxer64@qq.com; Jinxing Hu, hujinxing2000@163.com.

**Table S1**. Homologous proteins of different mycobacteria and Mab-EmbB and their protein identities

| Strain | homologous protein | identity |
| --- | --- | --- |
| Mtb | arabinosyltransferase EmbB | 66.91% |
| *M. bovis* | arabinosyltransferase | 67.60% |
| *M. canettii* | arabinosyltransferase EmbB | 68.24% |
| *M. marinum* | putative arabinosyltransferase B | 67.23% |
| *M. avium* | arabinosyltransferase EmbB | 67.79% |
| *M. ulcerans* | arabinosyltransferase EmbB | 67.15% |

**Table S2.** Primers used in gene editing

| **Primers** | **Sequences** |
| --- | --- |
| cr-embB-mut1-f | ***AT***GGCGTCGTCTTTGGCACCATCTTCT***A*** |
| cr-embB-mut1-r | ***AGCTT***AGAAGATGGTGCCAAAGACGACGCC***ATCT*** |
| cr-embB-mut2-f | ***AT***CCCCGCTTGGGGCCCGCTGTCGCAC***A*** |
| cr-embB-mut2-r | ***AGCTT***GTGCGACAGCGGGCCCCAAGCGGGG***ATCT*** |
| cr-embB mut3-f | ***AT***GGGCTGTTCGCCGCCCTGGGCGCCG***A*** |
| cr-embB mut3-r | ***AGCTT***CGGCGCCCAGGGCGGCGAACAGCCC***ATCT*** |
| cr-embB-mut4/5-f | ***AT***TAGTACCAGCCGAACGGGTCTTCCG***A*** |
| cr-embB-mut4/5-r | ***AGCTT***CGGAAGACCCGTTCGGCTGGTACTA***ATCT*** |
| sDNA-mut1-F | CCGCGGGCCGGCCTGGCGTGTGCTGGGCA**T**CGTCTTTGGCACCATCTTCTTCCTGATGT |
| sDNA-mut1-R | ACATCAGGAAGAAGATGGTGCCAAAGACG**A**TGCCCAGCACACGCCAGGCCGGCCCGCGG |
| sDNA-mut2-F | GCTTTTGATCAGCCGGGAAGTGTTGCCCG**G**CTTGGGGCCCGCTGTCGCACGCAGCAAGC |
| sDNA-mut2-R | GCTTGCTGCGTGCGACAGCGGGCCCCAAG**C**CGGGCAACACTTCCCGGCTGATCAAAAGC |
| sDNA-mut3-F | ACACCACTTCGGGCTGTTCGCCGCCCTGA**G**CGCCGCCGTGGCGGCGTTGGCCACGGTGC |
| sDNA-mut3-R | GCACCGTGGCCAACGCCGCCACGGCGGCG**C**TCAGGGCGGCGAACAGCCCGAAGTGGTGT |
| sDNA-mut4-F | CTACTTCCGCTGGTTCGGCAGCCCGGAAG**C**CCCGTTCGGCTGGTACTACAACCTGCTGG |
| sDNA-mut4-R | CCAGCAGGTTGTAGTACCAGCCGAACGGG**G**CTTCCGGGCTGCCGAACCAGCGGAAGTAG |
| sDNA-mut5-F | cagcaggttgtagtaccagccgaacgggt**T**ttccgggctgccgaaccagcggaagtagt |
| sDNA-mut5-R | actacttccgctggttcggcagcccggaa**A**acccgttcggctggtactacaacctgctg |

Note: The italicized and bolded letters indicate the sites of digestion, whereas the underlined and bolded letters denote the targeted mutation sites.

**(A)**


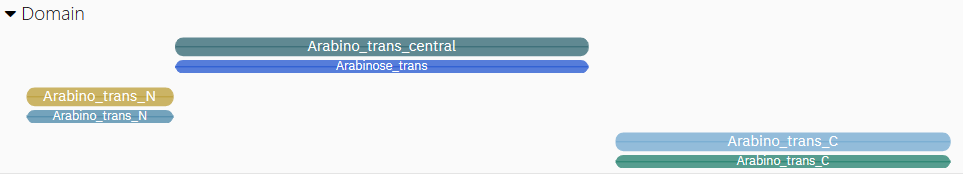


Pfam PF04602

Mycobacterial cell arabinan synthesis protein

209-666aa

**
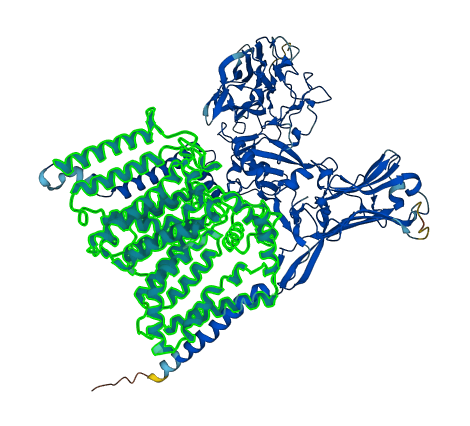
**

**(B)**

**Figure S1.** Protein structure of EmbB. **A)** InterPro predicts the function domain of EmbB. **B)** The protein structure forecasted by Alphafold. The green displays the central structural domain.

Mab_EmbB

Mab_EmbC

Consensus

Mab_EmbB

Mab_EmbC

Consensus

Mab_EmbB

Mab_EmbC

Consensus


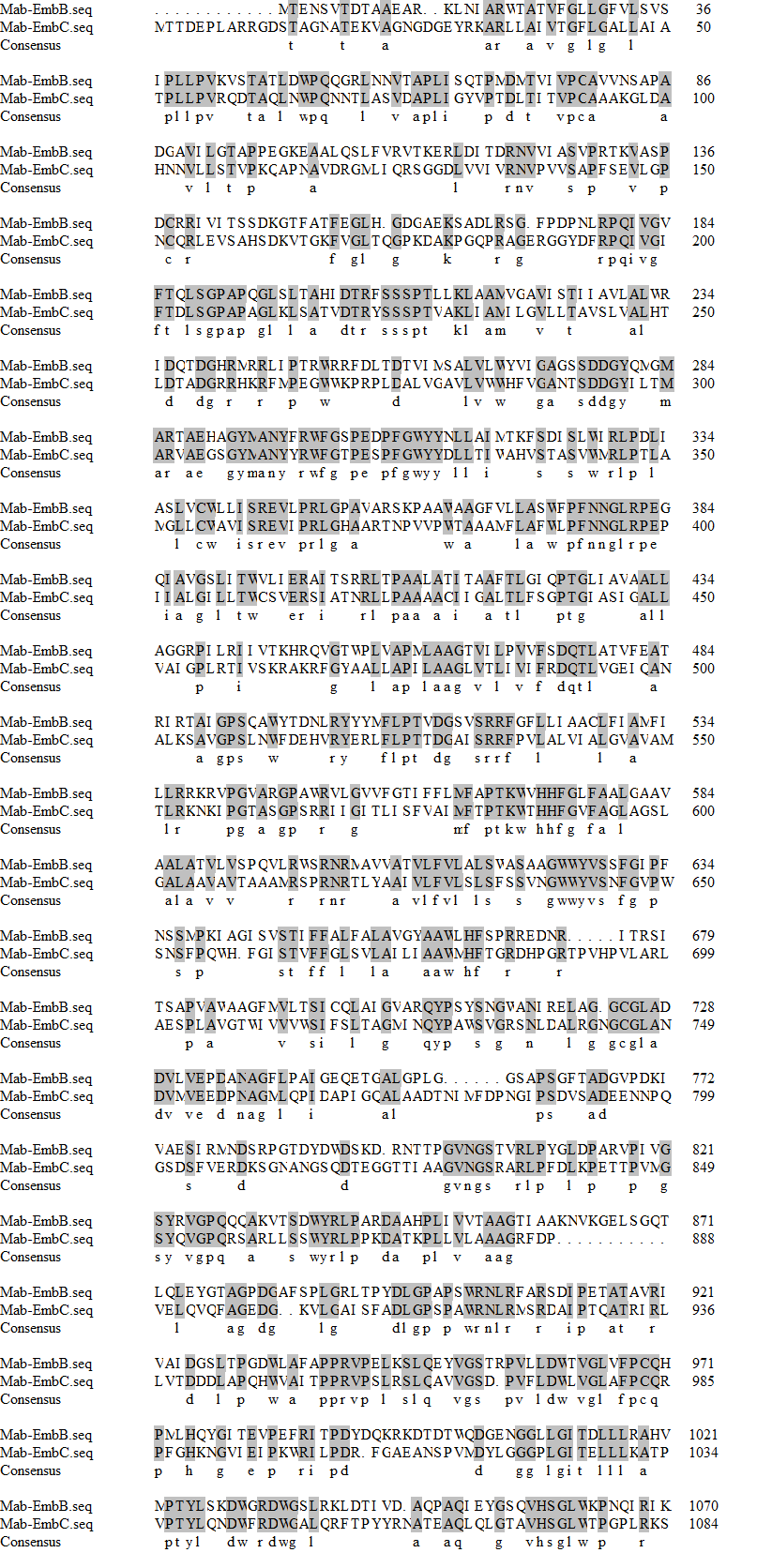

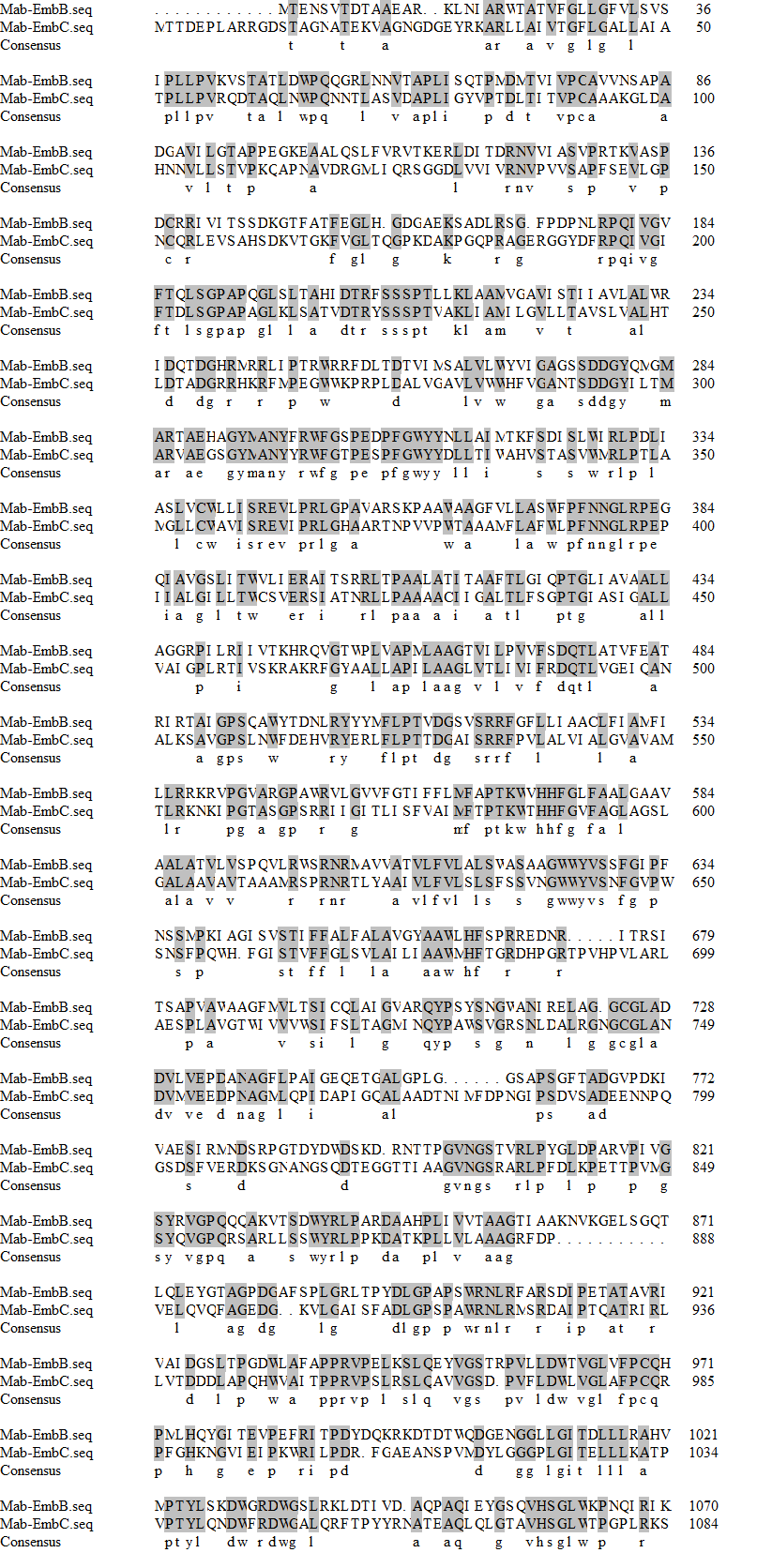


584

600

384

400

334

350

**Figure S2.** Partial results of Mab’s EmbB and EmbC proteins sequences alignment. The red boxes highlight the mutation sites.


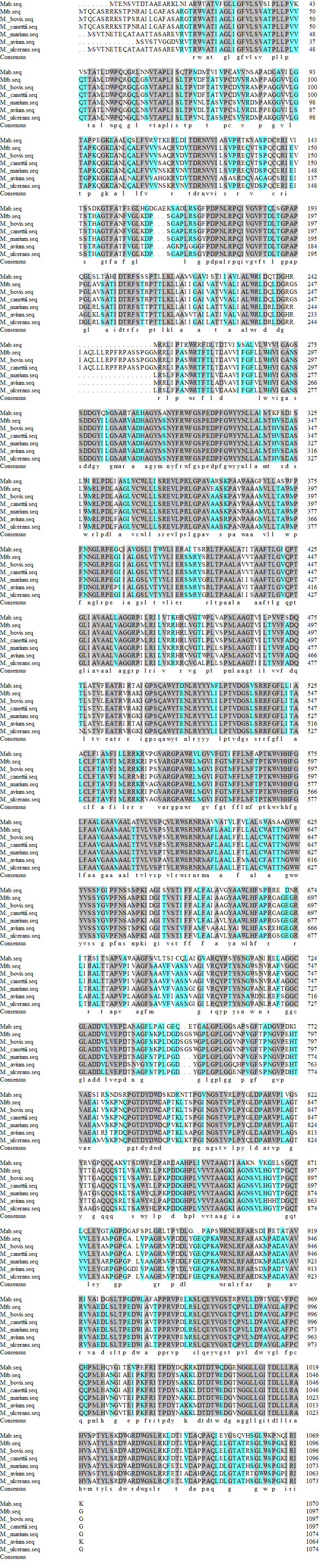

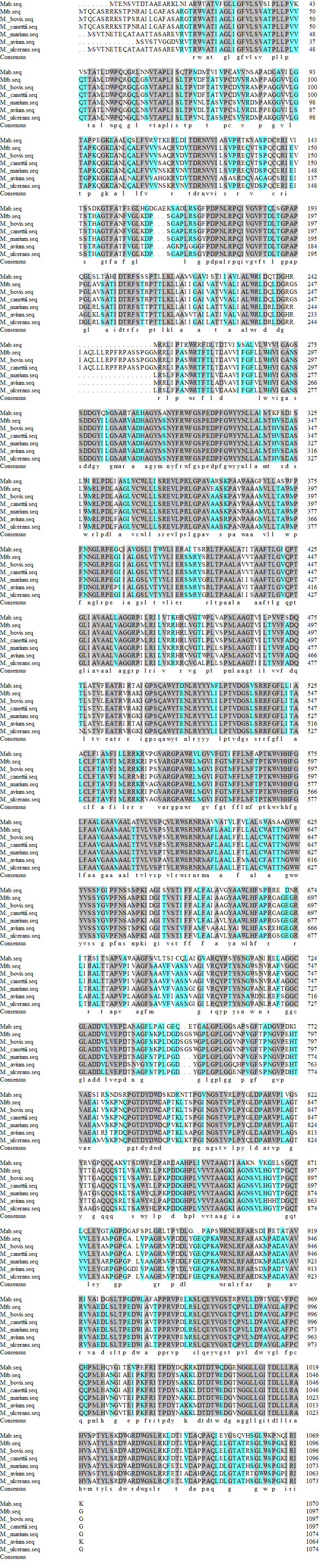


Mab

Mtb

*M. bovis*

*M.canettii*

*M. marium*

*M. avium*

*M.ulcerans*

Consensus

Mab

Mtb

*M. bovis*

*M.canettii*

*M. marium*

*M. avium*

*M.ulcerans*

Consensus

Mab

Mtb

*M. bovis*

*M.canettii*

*M. marium*

*M. avium*

*M.ulcerans*

Consensus

Mab

Mtb

*M. bovis*

*M.canettii*

*M. marium*

*M. avium*

*M.ulcerans*

Consensus

325

347

347

347

316

327

327

375

397

397

397

366

377

377

575

597

597

597

566

577

577

625

647

647

647

616

627

627

**Figure S3.** Partial sequence alignment of EmbB homologs in different mycobacteria and EmbB mutation positions of Mab. The red boxes highlight the mutation sites.
